# What do consumers with chronic conditions expect from their interactions with general practitioners? A qualitative study of Australian consumer and provider perspectives

**DOI:** 10.1111/hex.13050

**Published:** 2020-03-23

**Authors:** Hyun Jung Song, Sarah Dennis, Jean‐Frédéric Levesque, Mark Fort Harris

**Affiliations:** ^1^ Centre for Primary Health Care and Equity University of New South Wales Sydney NSW Australia; ^2^ Faculty of Health Sciences University of Sydney Sydney NSW Australia; ^3^ Ingham Institute for Applied Medical Research Liverpool NSW Australia; ^4^ Agency for Clinical Innovation Chatswood NSW Australia

**Keywords:** Australia, chronic disease, clinical interaction, expectations, general practitioners, patient experience

## Abstract

**Background:**

More than half of Australian adults manage one or more chronic conditions through ongoing interactions with general practitioners (GPs). Their experience of general practice interactions has important implications for their health outcomes and is thus important to explore in‐depth. Consumer expectations have emerged as a key consideration in this regard. How well they met in care settings can inform consumers' satisfaction and response to the care received. However, consumer expectations in Australian general practice are not well researched.

**Objective:**

To identify key consumer expectations in clinical interactions in Australian general practice based on consumer and GP perspectives.

**Design:**

Qualitative, phenomenological approach using thematic analysis of semi‐structured interviews.

**Setting and participants:**

Thirty‐one participants: 18 patients with one or more chronic (persisting > 6 months) conditions, 10 GPs and 3 GP registrars in Sydney, Australia.

**Results:**

Consumer expectations were strongly related to the context of their ongoing therapeutic relationship with a regular GP. Themes relating to some of the most commonly reported consumer expectations were as follows: (a) the importance of longevity and continuity; (b) having good rapport; (c) GP's respect for consumer opinions and expertise; (d) having effective communication; and (e) addressing mental health.

**Conclusion:**

Australian GPs and consumers prioritize a positive, long‐term clinical relationship in which they respect one another and can communicate their expectations freely**.** This has implications for consumer satisfaction and in turn ensuring relational continuity, which is particularly relevant to the ongoing care and management of consumers with chronic conditions.

## 1 INTRODUCTION

The burden of chronic conditions is a major problem in developed countries. Globally, close to three out of four older adults live with multiple chronic conditions.^1^ In Australia, half of the population live with at least one chronic disease, accounting for disproportionate health and cost burden.^2^ As in many other countries, much of the care and management of these individuals in Australia takes place in primary care settings, including general practice.^3^

General practitioners (GPs) are among various health professionals who play a vital role in the care of patients at this level. GPs act as the gatekeeper, manager and coordinator of various health services for patients, in addition to directly providing care that includes prevention and early intervention.^4^, ^5^, ^6^ They play a particularly important role in the care of individuals living with one or more chronic conditions, who require a multitude of services to manage their symptoms and illnesses. As such, these consumers tend to have regular and routine interactions with GPs, usually on a long‐term basis,^7^ and understanding their perception and experience of these interactions can provide a useful insight into the quality of care in general practice. The value of consumer‐reported quality of care has been widely documented in literature: consumers who report high quality of care have been shown to have better rates of treatment adherence,^8^, ^9^ improved self‐management skills,^10^ greater motivation (or ‘activation’) to manage care^11^ and a positive, ongoing relationship with their GP^12^ – factors that are critical to good chronic disease management. Thus, understanding what influences their perception of quality can help inform the delivery of more appropriate and effective care to these consumers.

Consumer expectations of care have been shown to be an effective predictor of perceived care quality.^13^, ^14^, ^15^ Juxtaposed against their actual experiences of care, these expectations – and whether or not they were met by the care provider – provide an indication of consumers' satisfaction with the care received.^14^, ^16^ This comparison forms the lens through which consumers perceive the quality of care and in turn can shape future expectations of care.^17^ The definition of consumer expectations varies in literature but is often understood as how likely consumers believe that ‘given events will occur during, or as an outcome of, health care’.^14^, ^18^ Consumer expectations have also been broadly conceptualized and expressed as beliefs, hopes, needs, wishes and desires about the health services they receive.^19^, ^20^ While expectations are partly about what consumers anticipate will happen (‘What is the care I think I *will* be getting?’), they also include their projection of what could happen under ideal circumstances (‘What is the care I think I *could* be getting?’).^14^, ^15^, ^21^ Consumer expectations can also be normative, in that they represent the consumers' evaluations of what they ought to or *should* receive from health services.^15^ Individuals managing chronic conditions usually have experience and knowledge of the health‐care system that enables them to speak on these types of expectations informatively. The views of such consumers are thus valuable to capture from research and clinical practice standpoints.

Despite the importance of understanding consumer expectations, there is a paucity of research in this area with respect to clinical interactions in Australian general practice. This knowledge gap presents a missed opportunity to understand the quality of ongoing clinical interactions for those managing chronic conditions. Through consumer and GP perspectives, our qualitative study thus aimed to identify key consumer expectations in clinical interactions, based on the experience of people that are managing one or more chronic conditions in Australian general practice. This work builds on a previous study to understand patient experience of general practice, which identified accessibility barriers leading up to the consultation, including the affordability of care and availability of various modes of service delivery.^7^

## 2 METHODS

A qualitative study was undertaken to capture and understand the breadth and depth of participant experiences. We conducted phenomenological research, which has been used in other studies to explore consumer expectations in health care.^19^, ^22^ This methodology is typically used to generate new knowledge about a phenomenon that is not well‐known or researched, so that others may learn about its ‘essential features’.^23^ Consistent with this approach, this study aimed to describe the *essence* – or common themes – of participants' lived experiences of general practice interactions.^24^, ^25^ While we attempted to describe these experiences as closely to how they were reported by the participants, we concurrently acknowledged that ‘there are no such things as uninterpreted phenomena’.^25^ In other words, the descriptions of participant experiences, including the themes that were identified, inevitably represent our own interpretation of these experiences. This was the underlying epistemological assumption in this study.

### 2.1 Sample and setting

Participants were recruited from three primary health networks (PHNs) in Sydney, Australia, including one rural area (Nepean Blue Mountains). A purposive sampling procedure was carried out to ensure that the cultural, linguistic, socio‐economic and geographic diversity of the Australian population was represented. Participants recruited were people living with one or more chronic conditions (persisting for more than 6 months)^26^ and GPs, including registrars. Consumers were initially recruited by an expression of interest through Health Consumers New South Wales. The inclusion criteria for consumer participants are described in Table 1. GPs were first recruited from practices known to the researchers and from directories provided by the PHNs. An invitation letter was sent by fax or email. Snowball sampling through relevant contacts known to participants completed the recruitment of all participants until thematic saturation was reached.

**TABLE 1:** Inclusion criteria for consumer participants

Inclusion criteria for consumers
Living with one or more chronic conditions (symptoms lasting ≥ 6 mo): With or without an official medical diagnosis Can be a rare, unknown or not well‐understood condition (self‐reported) Currently managing the condition(s) with a regular GP in general practice in one of three participating Sydney PHNs (Central and Eastern Sydney, South Western Sydney, Nepean Blue Mountains) At least 18 y of age Does not have a cognitive, psychological or other impairment that would prevent independent participation in the study.

### 2.2 Data collection

Qualitative, semi‐structured interviews were conducted between October 2016 and October 2017 by the researcher (HJS). Two separate and ‘mirrored’ interview guides were used for consumers and GPs. They were developed to ascertain (a) broad, descriptive information about the overall care journey in general practice and (b) participants' values and priorities with regard to specific aspects of the consumer experience, including clinical interactions. The guides were pilot‐tested with staff from the Centre for Primary Health Care and Equity, UNSW Australia, for ease of understanding and adapted as needed.

Interviews were held in person for GPs who requested this interview method for convenience and took place in their office. For consumers, interviews took place by telephone to allow participants to speak freely without social pressure (eg in the GP clinic) and due to distance. Interviews lasted between 15 and 60 minutes (mean of 26 minutes), were recorded and transcribed verbatim. Field notes were made by the interviewer during data collection. Participants were also invited to provide any additional responses or clarifications post‐interview by email or telephone.

### 2.3 Analysis

The data were coded and thematically analysed using a process described by Braun and Clarke.^27^ After the initial review and familiarization of the transcripts, the data were coded *inductively*, a process by which knowledge is derived from the data without using pre‐conceived conceptualizations or existing theories. Relevant and similar codes were then collated into themes, which were further refined using an iterative process in which themes and subthemes were added, removed, grouped, moved, relabelled, redefined and confirmed. Data analysis was done collaboratively by all researchers (HJS, SD, JFL, MH) and was guided by ongoing discussion of themes and iterative revisions of coding. Analytical memos were developed after coding and analysing each transcript, which were used to guide the interpretation of findings. NVivo 11 qualitative analysis software (QSR International) was used for coding, analysing and managing all data.

### 2.4 Ethics

This study was approved by the Human Research Ethics Committee at the University of New South Wales (HC16529). All participants were provided with a participant information sheet and provided their written informed consent to participate.

## 3 RESULTS

### 3.1 Participants

A total of 31 participants were included in the study, including 19 consumers and 13 GPs (including three registrars).

Information about participant characteristics was also collected at the time of the interview and is detailed in Tables 2 and 3. Consumers reported seeing the same GP for a median of 8 years. Four participants reported having a rare or poorly understood medical condition. More than half were recruited from patient advocacy or consumer representative organizations. Seven consumers also had a background as a health‐care professional or in a health care–related area (eg medical research, health‐care administration). Registered GPs (non‐registrars) in this study had been practising in general practice for a median of 26 years, and registrars had been practising for a median of 1.5 years.

**TABLE 2:** Characteristics of consumer participants

Participant characteristics	Number (% total or range, as indicated)
Sex	
Female	13 (72%)
Male	5 (28%)
Median age in years (range)	59.5 (29‐88)
Median years lived with condition(s) (range)	13 (1‐41)
Median years seeing current GP (range)	8 (3.5 mo‐17 y)
Presence of rare condition(s)	
Yes	4 (22.2%)
Recruited from patient advocacy or consumer representative organization	
Yes	11 (61.1%)
Has professional background or training in health care or related^a^ field	
Yes	7 (38.9%)

^a^ Health care–related field: no formal training as a health‐care or medical professional, but still within the health‐care field (eg practice staff, health‐care researcher).

**TABLE 3:** Characteristics of GP participants

Participant characteristics	Number (% total or range, as indicated)
Sex	
Female	6 (46%)
Male	7 (54%)
Number of	
GPs	10 (77%)
GP Registrars	3 (23%)
Median years working in general practice (range)	20 (2.5 mo‐50 y)
Median years working at current practice (range)	15 (1 wk‐30 y)
Work status	
Full‐time	7 (54%)
Part‐time	6 (46%)
Australian trained	
Yes	10 (77%)

### 3.2 Consumer expectations of general practice interactions

Consumer expectations were relayed through past and current experiences interacting with GPs and through participant beliefs on the factors that would constitute positive interactions during general practice consultations. GPs also reflected on their views on these expectations and provided their perspectives on how they may influence clinical interactions. Themes relating to some of the most commonly reported consumer expectations were as follows: (a) the importance of longevity and continuity; (b) having good rapport; (c) GP's respect for consumer opinions and expertise; (d) having effective communication; and (e) addressing mental health.

#### 3.2.1 The importance of relational longevity and continuity

##### Clinical benefits of informational continuity in chronic care

Participants reflected on the importance of having a regular, long‐term GP in managing their conditions. Consumers felt that this was integral to chronic care, as it enabled their GP to develop crucial contextual information about them over time, including a comprehensive overview of the individual's illness history, changes in symptoms, and health and non‐health needs:

[…] in the past I haven't necessarily had a regular GP and I can definitely see the benefit in having a regular GP who has an understanding and overview of your medical history, especially if you've had long term chronic illnesses. (Patient 1F03p)

GPs also perceived that the continuity in the relationship led to better health management and outcomes in consumers, especially for those with a complex set of health issues. Establishing this continuity with consumers was a priority for GPs, who stressed the importance of ensuring that they return routinely for visits:

Good quality health care involves establishing, number one, a regular GP. I think that for patients, particularly the ones that have complex chronic problems […] seeing just one person is the best way to have their health managed. I find that [those without a regular GP] have the most gaps versus the ones that just stay with one doctor. (GP Registrar 2F02)

Encouraging people to come regularly when they've got chronic disease I think is the thing we have to concentrate on the most. (GP 1F12)

##### Emotional aspects of having a long‐term therapeutic relationship

Consumers reported feeling reassured by having a GP who knew them well, as it removed the need to explain their condition and symptoms at each visit. This sentiment seemed to resonate strongly in those managing a rare or unknown condition, who reported having to frequently justify their illness to clinicians – particularly non‐specialists – who may not be familiar with their condition and set of symptoms. As one such consumer described, having a long‐term GP gave them a sense of reassurance and validation about their illness experience:

He said I don't have to prove anything to him because he's known me for a long time and knew me when I was healthy and active […] I guess that was very comforting because I have an invisible illness and I often have to prove it to people. Having that longevity and the fact that […] he knew enough about me. (Patient 1F01p)

Consumer participants further described a sense of closeness and comfort that developed from having a long relationship with their GPs, which they felt was a crucial part of their interactions:

[We] get along really well actually. I feel really comfortable that I can ask him anything, because I've known him for so long […] You kind of have a good patient/doctor relationship. (Patient 2F01p)

This was further emphasized by GP participants, who also valued relational longevity. They believed establishing a long‐term relationship led to an understanding between the consumer and GP that they acknowledged to be a priority for their patients:

[patients] get that feeling sometimes that only you understand them because you have been going on that journey for 15 years […] So, that's the longevity and I guess that privilege of being in the long journey and understanding them really well. So, it is this relationship I think is the most important thing that they value. (GP 1F13)

#### 3.2.2 Having good rapport

Participants described having a good rapport with the GP as another crucial aspect of the clinical relationship and a key component of consumer experience in general practice. The qualities consumers valued in GPs were their friendliness and professionalism that facilitated comfortable interactions:

The thing I like with him is that he's very personable […] I think it's really good that he has that relaxed, yeah, there's more of an interaction, which is what I get with [my GP]. It's great. (Patient 1M01p)

I mean he's very friendly but he's still very professional so I can certainly be very candid with him and there's a good rapport there. (Patient 1F03p)

However, there were some mixed views among consumers on the importance of having an informal, social rapport with their GP, with some participants valuing this aspect of their relationship (‘the GP and I have a rapport that we can talk about anything […] you can talk about your personal life’), while others placed little importance on this (‘I'm not there for a social chat’).

GP participants believed that having a positive relationship with consumers was crucial to their own work, with one participant characterizing general practice as ‘a relationship‐driven profession’. They perceived that this also enabled a greater sense of trust from consumers that made them more willing to agree with the GP's recommendations:

[…] have this good rapport and relationship with your patients, and they will follow what you're saying. (GP 1M07)

#### 3.2.3 Respect for consumer opinions and expertise

##### Consumers' confidence in their expertise

There were strong expectations from consumers in this study for GPs to respect their opinions and take them seriously. This appeared to be due to several reasons. Consumers tended to report strong confidence in their knowledge and capacity to manage their conditions. At the very minimum, all of the participants reported having acquired knowledge through the lived experience of managing a chronic condition, as well as from navigating and using various health services. This experiential knowledge appeared to facilitate a clear understanding of what they want out of their care:

[T]o be honest, I have been in and out of the hospital system for 17 years. I've had a trillion doctors and so I'm a really assertive patient and I generally know what I want and I have a pretty good understanding of what I want. (Patient 1M01p)

Consumers reported other characteristics and backgrounds that provided them with an additional source of knowledge. Examples of these qualities were having a professional background in health care (n = 7), connection to a consumer network (n = 11) and pursuing independent research. Those who seemed particularly compelled to seek information outside general practice were consumers managing conditions that are rare or not typically well understood in mainstream medicine (n = 4):

I work for a health care organisation, so we actually do a lot about patient‐centred care and all this stuff. I'm probably coming from a bit of high expectations maybe of what you should receive when you're sick […] (Patient 3F01p)

I've done enough research, particularly through Arthritis New South Wales, to only work with an evidence‐base. (Patient 2F03p)

Rare disease patients know a lot more than the qualified doctor does because they've research it to the nth degree. (Patient 2M01p)

Given this background, many consumers perceived having health literacy and confidence in their abilities. They felt that it was important for GPs to acknowledge and legitimize their expertise, especially as partners in the decision‐making process. Many expressed preferences for clinicians who did not enforce their own agenda, but instead respected consumer autonomy to make the final decision about their care:

He would just trust what I say in that we've done our own research and we want to see this specialist. (Patient 2F01p)

Our GP has said to us, “I strongly recommend that you have [the treatment],” but she hasn't said, “You must have it.” She said, “Its' your decision.” […] I think that relationship is really good […] (Patient 2F03p)

Moreover, consumers appreciated their GP's ability to admit when they did not know something, which they perceived as a demonstration of humility that reinforced the strength of their relationship:

[…] the reason why I go back to her is because she is willing to say, “We don't know.” Or, “We're not sure yet. It hasn't been researched. (Patient 1F04p)

#### 3.2.4 Effective communication

Good communication was viewed by consumers and GP participants as the cornerstone of the therapeutic relationship. In this sense, communication comprised of more than the exchange of health information, but rather, it was about feeling comfortable enough with their GP to ask questions, raise concerns and complaints, and feeling listened to without feeling dismissed. This was important given the complex and dynamic nature of managing long‐term conditions:

Because I've got so many different medical issues going on, I think it is important that you feel relaxed with them and you trust them and you can ask them anything […] (Patient 2F01p)

Part of good communication in a clinical setting was also described by consumers as being able to disagree with the GP's recommendation without fear of judgement or criticism:

I would again feel comfortable saying to her, ‘No, I actually don't want to follow up with that recommendation’ and I wouldn't feel judged or criticised for doing that. (Patient 1F05p)

Non‐verbal communication was also viewed as an essential part of good communication, and this related to the GP knowing consumers well enough to pick up on non‐verbal cues and reading body language, including in situations where they were not capable of expressing themselves verbally:

[…] because I know them, at times I am able to pick up an expression on their face where I'll say, “Something I said worries you,” or, “Did you really understand what I was saying?” That's something that as a doctor, as a health care professional I have to be aware of. I have to try and read body language as well to see whether in fact my communication is getting through.” (GP 3M05)

Effective communication also appeared to be facilitated by the GP's ability to be *active* listeners. GP participants described this as listening attentively and following up with reassurance of consumers' concerns and fears. GPs felt this approach helped to alleviate fears and anxiety in consumers, which they believed was an important aspect of their role as care providers:

I listen to them […] unless you address their fears, it is not good medicine. […] because that's important for patients. They go away and they feel a load is off my back just because the doctor listened to me, talked and explained things to me. (GP 1M07)

#### 3.2.5 Addressing consumer mental health

Mental health issues were discussed often in this cohort and were reported to impact significantly on the overall patient experience. Several consumers described mental health issues as a significant challenge that arose from the difficulties of managing a chronic condition (‘It's a very significant part of my condition’.) and reported expectations for the GP to ask about their mental health as part of routine care. Thus, a GP's initiative to ‘check in’ with their patients about their mental health was viewed positively by consumers. One participant related this to her GP's empathetic and caring nature:

She has oodles of empathy and always puts my mental health front and centre and makes sure, apart from managing physical symptoms, that she's always checking in with how I'm doing from a mental health perspective. (Patient 1F05p)

Some consumers perceived that looking after the patient's mental health was a marker of the GP's competence, including their ability to be thorough and intuitive clinicians. They expected GPs to address mental health issues – in addition to the physical chronic condition – as a part of delivering whole‐person care:

He doesn't just treat the condition that I'm coming in for. He'll ask me about my stress levels and anxiety levels and mental health. He's quite intuitive, I think […] he's really good. (Patient 1F03p)

Mental health is something that is not looked after […] There's not this overall looking after your health, your whole body. It's just this part and that part […] but not looking after the whole person. (Patient 2M01p)

## 4 DISCUSSION

### 4.1 Summary of findings

In this study, consumer expectations were based strongly on the context of their ongoing therapeutic relationship with a regular GP. These expectations were as follows: maintaining a long‐term relationship with the same GP, having good rapport, being respected for their knowledge and opinion, having effective communication and having their mental health addressed.

### 4.2 Comparison to other research and practical implications

Participants identified that the consumer–GP relationship, in which expectations are communicated and responded to appropriately, is important to consumer experience of clinical interactions in general practice. For those with chronic conditions, these experiences were not necessarily based on individual clinical interactions, but in the context of an ongoing relationship with the same GP. This is not surprising given that the management of chronic conditions is often a long‐term endeavour shared with the GP.^4^, ^7^

Relational continuity appeared to be related to and reinforced by other interpersonal expectations. For instance, having the same GP contributed to a sense of trust and comfort that enhanced the quality of clinical rapport and of interpersonal communication. This expands on previous literature about the importance of sustained therapeutic relationships to the care of those with chronic conditions.^28^, ^29^, ^30^, ^31^ In our study, GPs reported that relational and informational continuity facilitated more effective consultations, as well as better overall management of patients. Previous research has similarly shown that continuity of care helps GPs perform clinical tasks more effectively, including improving the diagnosis and management of chronic and complex conditions.^30^, ^31^ In this study, relational continuity also enabled GPs to develop a deeper understanding of their patients as individuals with multifaceted needs, which is often viewed as being crucial to the delivery of patient‐centred chronic care.^32^, ^33^

Ongoing interactions with the same GP have important implications for shared decision‐making. By becoming more familiar with each other over time, consumers and GPs are able to negotiate how their expectations and priorities will be met during clinical interactions. We found that this not only gives consumers an idea of what to expect from current and future interactions, but it also enables them to feel comfortable enough with their GPs to express them openly. The links between therapeutic relationships, development of expectations and effective communication are integral to patient management and deserve further exploration in research.^34^, ^35^

Feeling respected for their expertise and opinions was another important consumer priority that was identified in the study. Consumers discussed their confidence in their perceived health and health‐care literacy to navigate and use services effectively. Literature supports the notion that health literacy is a ‘generative’ skill, or an ongoing process that develops over time through a multitude of experiences and encounters throughout the care journey.^36^, ^37^ In line with this thinking, consumers develop greater knowledge and skills through the experience of managing long‐term conditions. In this study, input from such experienced consumers was valued by GPs in ensuring good quality of care for collaborative decision‐making (‘good quality of care is a compromise between the patients' and doctors' agenda’) and for overall service improvement (‘you can't do quality improvement without understanding what their experience is […] they bring a very good, different perspective’). While our GP participants acknowledged the value of seeking consumer knowledge, the collection and use of consumer feedback are not formalized in Australian general practice, highlighting a need to address this gap in both clinical practice and at a policy level.^38^

Consumers also raised the importance of addressing mental health needs in addition to their chronic physical illness. The strong association between chronic physical illness and depression and anxiety has been documented in literature.^39^, ^40^, ^41^ The association goes both ways: depression and anxiety are more common in those with a chronic physical illness than the general population^39^; in turn, the presence of mental illness exacerbates the negative outcomes of chronic conditions such as functional outcomes, quality of life and increased mortality.^41^, ^42^, ^43^ In order to alleviate this burden on patients and improve outcomes, chronic disease management should incorporate the provision of mental health care, including assessment, planning and review at the general practice level.^44^ As expressed by our participants, routinely looking after their mental health may be an important way to address consumer expectations around a holistic, whole‐person approach to chronic care.

Consumers in Australia are not required to stay with the same GP.^28^ They have the freedom to change GPs easily if they are not satisfied with their care, although initiatives such as Health Care Homes are currently being trialled to enrol and manage people with complex needs within a single practice.^45^ Our findings show that GPs endeavour to negotiate with and manage consumer expectations partly to ensure they return. This is relevant because for those living with chronic conditions, continuity of care with a GP can lead to better clinical outcomes,^46^, ^47^ more effective coordination of care^48^ and increased patient satisfaction.^49^ While seeing the same GP may not be a priority for all consumers, especially those with more acute needs,^12^ relational continuity with a trusted GP is important to consumers managing chronic and complex problems.^30^ One could assume that those whose expectations are not met are not likely to return to the same GP. It would be useful to research this relationship in future studies, especially in the Australian context where patients can easily change GPs if they are not satisfied.

### 4.3 Study limitations and areas for future research

Our participant cohort comprised of a subset of consumers, many of whom had backgrounds and experiences that provided them with high self‐reported health literacy and strong motivation to manage their care. For instance, more than half of the consumers in the study had a professional or personal connection to the health‐care profession, and several had unique knowledge as patients with a rare or unknown condition. Many were also recruited through consumer organizations. This high level of health literacy and confidence in their knowledge likely informed their high expectations of care,^50^ which may not be representative of the general population.

Many of the GPs who were included in the study also represented a unique subset of their peers due to the way they were recruited. Initially, GPs who were known to the researchers were invited to participate. These individuals were clinicians who had previously participated in or expressed interest in research activities in improving patient care. Some of the GP participants were also actively involved in the area as clinician‐researchers or they had academic appointments and were also GP educators. Further snowball sampling of GPs through the participants' own contacts completed the recruitment process. For those who were not recruited through a research‐related network (eg through the PHN directories), there was still a self‐selection of clinicians who were interested in the topic, since there were no other incentives provided for participation. As such, and as reflected in the richness of the interview data, these participants seemed well‐versed and keenly motivated to learn more about patient‐centred care in general practice. This was both a strength and limitation because while this may limit our ability to generalize study findings, we were able to capture the rich perspectives of those with a strong interest in the research topic. This is important for a study like ours which is the first of its kind to explore this topic in depth.

Additionally, despite efforts to recruit from culturally and linguistically diverse regions of Sydney, this level of diversity was not reflected in our study sample, particularly in our patient cohort. We surmise that this may have been partly due to the English language requirements of the study inclusion criteria. Exposure to other health‐care systems and traditions, as well as varying levels of health and health‐care literacy, likely influences expectations of care,^18^ as such, it would be interesting to study and compare the unique perspectives of these consumers. Furthermore, there may be those who are unable or unwilling to express their expectations during the clinical exchange, even in the context of long‐term relationships with their GP. Understanding how cultural and other factors influence the expression of their expectations during consultations would be extremely valuable to explore in future research.

Finally, because of this study focused on people with chronic conditions with a regular GP (ie median duration of seeing same GP was 8 years), the findings may not be transferable in the context of those with more acute needs and without a regular GP. The strong emphasis on relational continuity may be less significant to this group of consumers.

## 5 CONCLUSION

This study found that for consumers managing a chronic condition, expectations of general practice interactions were heavily based on their ongoing relationship with a regular GP. Within this context, GPs and consumers prioritized a continued, long‐term relationship which facilitated positive rapport, respected consumer expertise, effective communication and attention to consumer's mental health. An important area for future research would be to test these expectations and see how they are being met in general practice settings. These findings may inform the way that GPs engage and make decisions with chronic disease patients. We recommend that assessing consumer expectations needs to be a crucial component of evaluating their general practice experience.

## CONFLICT OF INTEREST

The authors declare no conflicts of interest.

## Data Availability

The data that support the findings of this study are available on request from the corresponding author. The data are not publicly available due to privacy or ethical restrictions.
